# Long-term outcomes of pace-and-ablate strategy in patients with atrial fibrillation

**DOI:** 10.1007/s10840-025-02038-3

**Published:** 2025-04-07

**Authors:** Johan van Koll, Madelon D. E. A. Engels, Jesse H. J. Rijks, Madelon Salari, Jelle Luijten, Joost Lumens, Vanessa P. M. van Empel, Sjoerd W. Westra, Antonius M. W. van Stipdonk, Theo A. R. Lankveld, Sevasti M. Chaldoupi, Jacqueline Joza, Rypko J. Beukema, Justin G. L. M. Luermans, Dominik K. Linz, Kevin Vernooy

**Affiliations:** 1 Department of Cardiology, Cardiovascular Research Institute Maastricht (CARIM), Maastricht University Medical Center, Maastricht, The Netherlands; 2 Department of Cardiology, Radboud University Medical Center, Nijmegen, The Netherlands; 3 Department of Biomedical Engineering, Cardiovascular Research Institute Maastricht (CARIM), Maastricht University Medical Center, Maastricht, The Netherlands; 4 Department of Medicine, Mcgill University Health Center, Montreal, QC Canada

**Keywords:** Atrial fibrillation, Atrioventricular node ablation, Right ventricular pacing, Cardiac resynchronization therapy, Heart failure, Survival analysis

## Abstract

**Background:**

The pace-and-ablate strategy is second -line therapy to obtain rate control in patients with persistent symptomatic atrial fibrillation (AF) when other treatment options fail. This study aims to evaluate long-term effects on clinical outcomes following pace-and-ablate strategy in AF patients.

**Methods:**

This retrospective study includes patients who underwent successful pacemaker implantation (right ventricular pacing (RVP) or cardiac re-synchronization therapy (CRT)) followed by atrioventricular node ablation (AVNA) between 2010 and 2020. Patients were treated according to the prevailing guidelines. The primary endpoint was a composite of all-cause mortality and heart failure hospitalization (HFH). Secondary endpoints were individual outcomes of all-cause mortality, HFH, and left-ventricular ejection fraction (LVEF) change.

**Results:**

Two hundred ninety-eight patients were included, 162 undergoing RVP, and 136 receiving CRT, with a median follow-up of 5.8 years [4.1–8.0]. The primary endpoint occured in 47% of the RVP group and 49% of the CRT group (*p* = 0.206). All-cause mortality occurred in 36% of the RVP group and in 45% of the CRT group (*p* = 0.005). HFH occurred in 22% of the RVP group and in 15% of the CRT group (*p* = 0.328), with 17(10%) upgrades to CRT in the RVP group. Median LVEF in the RVP group remained stable (56% [49–60] to 53% [43–57]; *p* = 0.081), while it improved in the CRT group (31% [22–38] to 43% [32–51]; *p* < 0.001).

**Conclusion:**

Mortality and HFH in patients with AF managed through a pace-and-ablate strategy are high. Reassuringly, LVEF deterioration requiring upgrade to CRT is uncommon in patients undergoing RVP with normal baseline LVEF before AVNA. CRT improves LVEF in patients with reduced LVEF before AVNA.

**Graphical Abstract:**

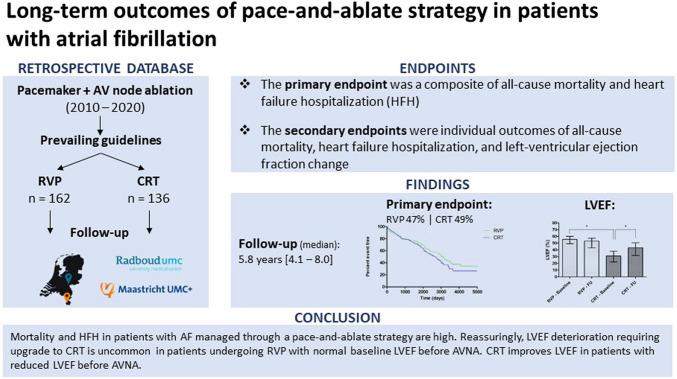

## Introduction

Atrial fibrillation (AF) is associated with a high symptom burden, reduced quality of life and increased risk of death and a wide range of morbidities including heart failure (HF), often due to tachycardia-induced cardiomyopathy (TIC), which has a considerable public health burden [1–4]. For patients unresponsive or intolerant to intensive (pharmacological) therapy and unsuitable for rhythm-control by catheter ablation, permanent pacemaker implantation with subsequent atrioventricular node ablation (AVNA), a so-called pace-and-ablate strategy, is a frequently used alternate treatment [5]. This strategy is considered safe with good symptom reduction, improvement in quality of life, and has shown to improve cardiac function in selected populations [4, 6]. Additionally, AVNA should be considered in patients with AF and insufficient biventricular pacing (< 90–95%) or in those experiencing inappropriate implantable cardioverter-defibrillator (ICD)- or cardiac resynchronization therapy (CRT)-D shock therapy due to AF [7–9]. Within the pace-and-ablate strategy, the preferred pacing modality mainly differs depending on the patient’s left ventricular ejection fraction (LVEF) prior to AVNA [7]. However, in some patients with normal LVEF, chronic right ventricular pacing (RVP) may lead to adverse cardiac remodeling and potentially to HF, a condition referred to as pacing-induced cardiomyopathy (PICM) [10]. In these patients, an upgrade from RVP to CRT should be considered [7].

The long-term outcomes of patients with AF managed using the pace-and-ablate strategy is currently unclear, and long-term clinical follow-up is sparse [3, 11]. This retrospective study aims to fill this gap by investigating the long-term effects of a pace-and-ablate strategy on clinical outcomes and left-ventricular function in patients receiving either RVP or CRT.

## Materials and methods

This is a retrospective, dual-center, observational study performed at the Maastricht University Medical Center + (MUMC + , Maastricht, the Netherlands) and Radboud University Medical Center (Radboudumc, Nijmegen, the Netherlands), evaluating the long-term effect of a pace-and-ablate strategy on clinical outcomes. This study was approved by the local ethics board of both institutions and regarded non-WMO. The research adhered to the Declaration of Helsinki.

### Patient selection

All patients who underwent AVNA between 2010 and 2020 were screened for eligibility. Patients were included if a successful AVNA was performed post RVP or CRT. Indications for AVNA included: refractory AF symptoms, refractory pharmacological rate control, optimization of biventricular-pacing, ventricular response rate in the shock-zone of an ICD, rapid clinical worsening of TIC. The only exclusion criterion was a written objection to the utilization of patient data for scientific research, as noted in the electronic patient file. All available follow-up data were collected for this study.

### Primary endpoint

The primary endpoint was the first occurrence of a composite of all-cause mortality and HF hospitalization (HFH) during follow-up. HFH was defined as unplanned hospitalization of more than 1 day due to signs and symptoms consistent with congestive HF or upgrade from RVP to CRT due to PICM.

### Secondary endpoints

Secondary endpoints were time-to-event analyses of the individual components of the primary endpoint. Furthermore, changes in LVEF and predictors for the development of PICM were analyzed.

### Baseline characteristics

Baseline characteristics were collected using the locally available electronic patient record. The LVEF as assessed by last transthoracic echocardiogram (TTE) performed within 1 year prior to AVNA was considered the baseline measurement.

### Procedure

The date of device implantation or upgrade prior to AVNA was recorded, together with type of pacing (RVP or CRT) and type of device (transvenous pacemaker, leadless pacemaker, transvenous ICD or CRT-D/P). Additionally, the date and indication for AVNA were collected. The decision between RVP and CRT device implantation was based on the guidelines at that time. These guidelines recommended RVP for patients with preserved LVEF, while CRT was recommended for those with reduced LVEF.

### Follow up

Data collection was performed until July 1st 2024. All-cause mortality, HFH and upgrades to CRT were collected until end of follow-up. Changes in LVEF, based on TTE measurements between baseline and follow-up, were assessed. The follow-up TTE was either the most recent TTE or the last TTE before device upgrade (e.g., from RVP to CRT) was performed. Duration of follow up was calculated as date of AVNA to July 1st 2024 or death. All data and follow-up dates were censored after July 1st 2024.

### Statistical analysis

Descriptive continuous data were reported as mean (± standard deviation (SD)) or median (interquartile range (IQR)), depending on the distribution of the data. Categorical data were summarized using frequencies (*n*) and percentages (%). Descriptive statistics were reported for the full study population and stratified by RVP and CRT. Comparison between the RVP and CRT group was accomplished by using the chi-square statistics or Fisher’s exact test for categorical data, and the independent samples *T*-test or Mann–Whitney *U* test as appropriate. Within group analysis was performed using the paired-samples *T*-test or Wilcoxon Signed rank test depending on the distribution of the data.

Survival analysis for both the RVP and CRT group was performed using Kaplan–Meier survival analysis and compared for statistical differences using the Log-Rank test. For survival curves, time censoring was determined by time to first event or time to censoring. Hazard ratios (HR) were calculated for both groups using the Cox proportional hazards regression model.

All statistical analyses were performed using IBM SPSS Statistical Software version 28 (SPSS Inc.). In all statistical analyses, a *p*-value of < 0.05 was considered statistically significant.

## Results

### Baseline characteristics

This dual-center study included 298 patients. The patient characteristics are summarized in Table 1. Among them, 162 patients (54%) underwent RVP while 136 patients (46%) received CRT. From the study population, the age was 71 ± 10 years with a nearly equal sex distribution, and a BMI of 27.4 ± 5.6 kg/m^2^. Paroxysmal AF was the predominant type of AF in this cohort (35%), followed by persistent AF (30%) and permanent AF (28%); the type of AF was unknown for 24 patients (8%). AVNA was performed in most patients (77%) to reduce AF symptom burden; different indications are described in Table 1.

**Table 1:** Baseline characteristics

Characteristics	Total population (*n* = 298)	RVP (*n* = 162)	CRT (*n* = 136)	*p-*value
Female sex, *n* (%)	141 (47)	101 (62)	40 (29)	< 0.01
Age (years), mean SD	71 ± 10	72 ± 9	68 ± 10	< 0.001
BMI (kg/m^2^), mean SD	27.5 ± 5.6	26.9 ± 5.3	28.3 ± 5.9	0.037
*Comorbidities*				
Prior cardiac surgery, *n* (%)	77 (26)	42 (26)	35 (26)	0.97
Prior invasive treatment for arrhythmia, *n* (%)	85 (29)	55 (34)	30 (22)	0.024
Coronary artery disease, *n* (%)	87 (29)	35 (22)	52 (38)	0.005
Stroke or TIA, *n* (%)	46 (15)	32 (20)	14 (10)	0.024
Diabetes mellitus, *n* (%)	52 (17)	18 (11)	34 (25)	0.002
Hypertension, *n* (%)	147 (49)	87 (54)	60 (44)	0.099
Thyroid disease, *n* (%)	41 (14)	25 (15)	16 (12)	0.632
COPD, *n* (%)	33 (11)	16 (10)	17 (13)	0.472
OSA, *n* (%)	36 (12)	17 (10)	19 (14)	0.359
Malignancy, *n* (%)	46 (15)	30 (19)	16 (12)	0.108
*Type of AF*				0.002
Paroxysmal, *n* (%)	103 (34.56)	70 (43.21)	33 (24.26)	
Persistent, *n* (%)	88 (29.53)	44 (27.16)	44 (32.35)	
Permanent, *n* (%)	83 (27.85)	36 (22.22)	47 (34.56)	
Unknown, *n* (%)	24 (8.05)	12 (7.41)	12 (8.82)	
*Device type*				
RVP, *n* (%)	147 (49)	147 (91)	-	
Leadless device, *n* (%)	4 (1)	4 (2)	-	
Single-chamber ICD, *n* (%)	11 (4)	11 (7)	-	
CRT-P, *n* (%)	42 (14)	-	42 (31)	
CRT-D, *n* (%)	94 (32)	-	94 (69)	
*Reason AVNA*				
Symptomatic AF, *n* (%)	229 (77)	153 (94)	76 (56)	
Optimization biventricular pacing, *n* (%)	48 (16)	-	48 (35)	
AF with RVR into shock-zone, *n* (%)	15 (5)	7 (4)	8 (6)	
Clinical worsening TIC, *n* (%)	6 (2)	2 (1)	4 (3)	
*Echocardiography*				
Baseline LVEF (%), median IQR	-	56 [49–60]	31 [22–38]	< 0.001

*AF;* atrial fibrillation, *AT;* atrial tachycardia, *BMI;* body-mass index, *COPD;* chronic obstructive pulmonary disease, *CRT;* cardiac resynchronization therapy, *ICD;* internal cardioverter-defibrillator, *OSA;* obstructive sleep apnea, *RVP;* right ventricular pacing, *RVR;* rapid ventricular rate, *TIA;* transient ischemic accident, *TIC;* tachycardia-induced cardiomyopathy

In the RVP group, the mean age was 72 ± 9 years, 62% were female, median LVEF was 56% [49–60], and the mean BMI was 26.9 ± 5.3 kg/m^2^. Paroxysmal AF was the predominant type of AF in (43%), followed by persistent AF (27%), permanent AF (22%), with 8% of patients having an unknown type of AF. A total of 55 patients (34%) had received prior AF ablation. A total of 147 patients (91%) received a standard pacemaker, 11 patients (7%) received an ICD, and four patients (2%) were treated with a leadless pacemaker. In most patients (94%), AVNA was performed to treat symptomatic AF. AVNA was performed in seven patients (4%) due to AF with a ventricular response rate in the shock-zone of the ICD, and in two patients (1%) because of rapid clinical worsening due to suspected TIC.

In the CRT group, the mean age was 68 ± 10 years, 29% were female, median LVEF was 31% [22–38], and the mean BMI was 28.3 ± 5.9 kg/m^2^. Permanent AF was the predominant type of AF in the CRT group (35%), followed by persistent AF (32%), and paroxysmal AF (24%). In 9% of patients, type of AF was unknown. A total of 30 patients (22%) had received prior AF ablation for their arrhythmia. Most patients (*n* = 94; 69%) in the CRT group were treated with a CRT-D device. Seventy-six patients (56%) in the CRT group underwent AVNA to treat symptomatic AF, and AVNA was performed in 48 patients (35%) to optimize biventricular pacing, eight patients (6%) because of AF with a ventricular response rate in the shock-zone of the CRT-D, and in four patients (3%) because of rapid clinical worsening due to suspected TIC.

Baseline characteristics differed between the RVP and CRT groups (Table 1). Patients in the CRT group were younger, more often male, and had a higher BMI compared to the RVP group. Fewer patients in the CRT group had undergone prior ablation for atrial arrhythmia (22% vs 34%), while more were previously treated for- or diagnosed with coronary artery disease (CAD) (38% vs 22%). Also, fewer patients in the CRT group had a history of stroke or transient ischemic attack (TIA) (10% vs 20%), while more were diagnosed with diabetes mellitus (DM) (25% vs 11%). Paroxysmal AF was more common in the RVP group as compared to the CRT group (43% vs 24%).

### Clinical outcome

**Fig. 1:**
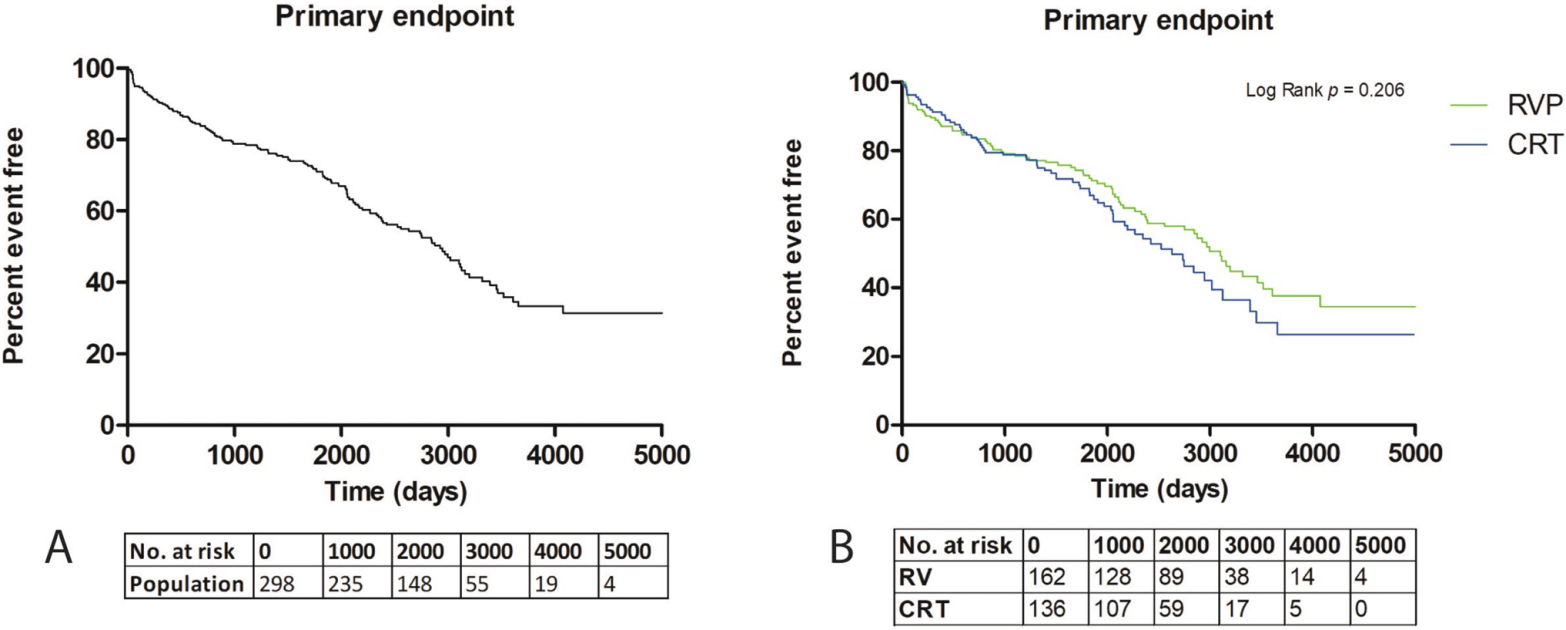
**A** Kaplan–Meier survival curve of time to primary endpoint in total study population. **B** Kaplan–Meier survival curve of time to primary endpoint in patients with right ventricular pacing and cardiac resynchronization therapy. CRT, cardiac resynchronization therapy; HFH, heart failure hospitalization; RVP, right ventricular pacing

**Fig. 2:**
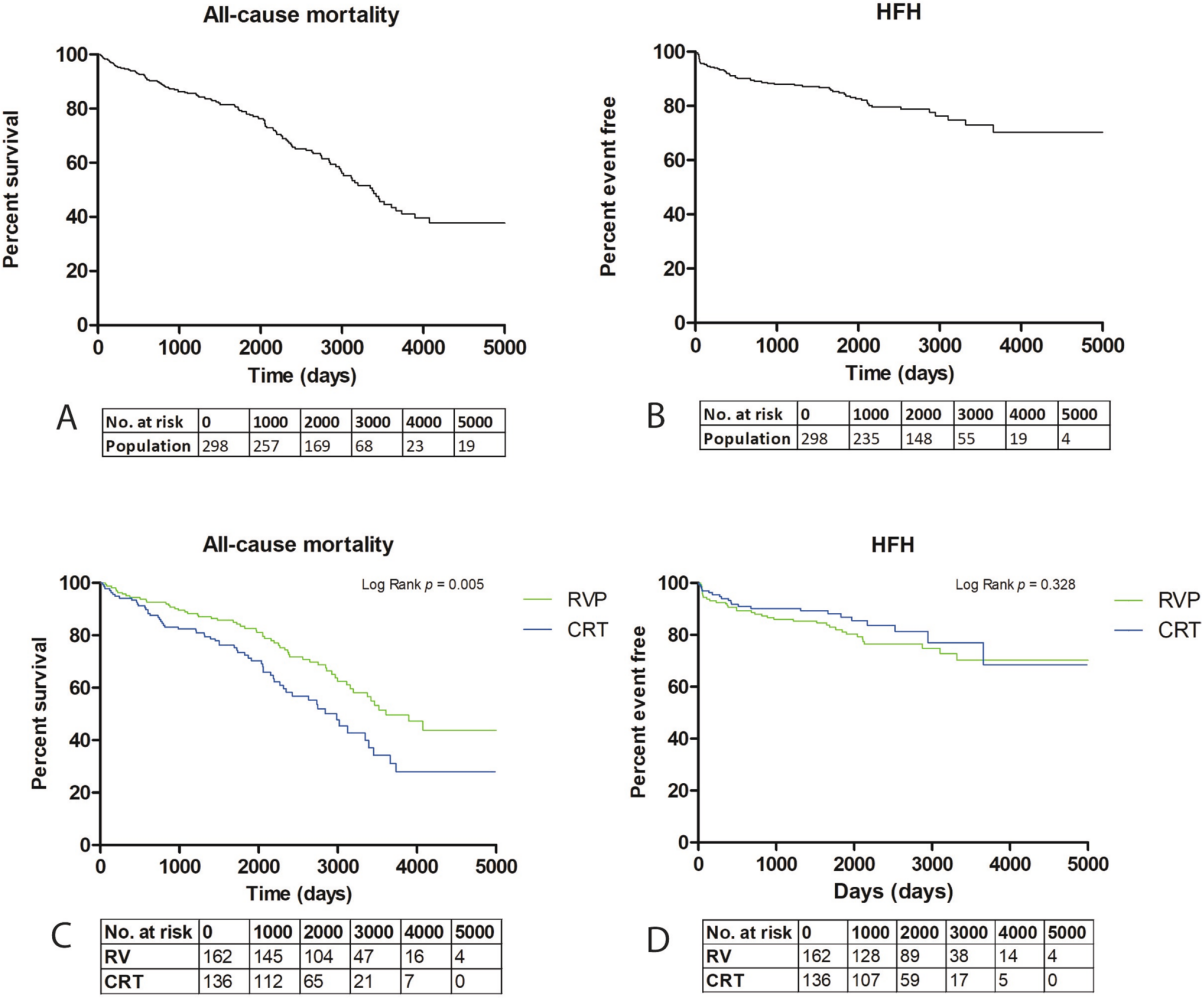
**A** Kaplan–Meier survival curve of time to all-cause mortality in total study population. **B** Kaplan–Meier survival curve of time to heart failure hospitalization in total study population. **C** Kaplan–Meier survival curve of time to all-cause mortality for patients with right ventricular pacing and cardiac resynchronization therapy. **D** Kaplan–Meier survival curve of time to heart failure hospitalization for patients with RVP or CRT. CRT, cardiac resynchronization therapy; HFH, heart failure hospitalization; RVP, right ventricular pacing

The primary endpoint, which was a composite of all-cause mortality and HFH, was observed in 142 patients (48%) with a median follow-up time of 5.8 years [4.1–8.0] (Fig. 1A). The primary endpoint was mainly driven by mortality, which was observed in 119 patients (40%) (Fig. 2A), whereas HFH was observed in 57 patients (19%) (Fig. 2B).

In the RVP group, the primary endpoint was observed in 76 patients (47%) with a median follow-up time of 6.6 years [4.4–8.7] (Table 2). In this group, the clinical endpoint of all-cause mortality occurred in 58 patients (36%) and HFH in 36 patients (22%). Seventeen patients (10%) underwent an upgrade from RVP to CRT because of worsening HF or PICM.

**Table 2:** Comparison of primary endpoint and separate analysis between right ventricular pacing and cardiac resynchronization therapy

	Population (*n*=298)	RVP (*n*=162)	CRT (*n*=136)	HR	95% CI
Combined endpoint					
* All-cause mortality; HFH; n (%)*	142 (48)	76 (47)	66 (49)	1.24	0.89–1.73
Separate endpoint analysis					
* All-cause mortality, n (%)*	119 (40)	58 (36)	61 (45)	1.67	1.16–2.40
* HFH, n (%)*	57 (19)	36 (22)	21 (15)	0.76	0.45–1.31

*CI;* confidence interval, *CRT;* cardiac resynchronization therapy, *HFH;* heart failure hospitalization, *HR;* hazard ratio, *RVP;* right ventricular pacing

In the CRT group, the primary endpoint occurred in 66 patients (49%), with a median follow-up time of 5.2 years [3.9–7.4] (Table 2). The clinical endpoint of all-cause mortality occurred in 61 patients (45%), and HFH occurred in 21 patients (15%) in the CRT group.

Interestingly, comparison between both groups showed no statistically significant difference in the occurrence of the primary endpoint (HR 1.24 (95% CI, 0.89–1.73)). Kaplan–Meier estimates of event-free survival showed no significant separation of the event-free survival curves of RVP and CRT (*p* = 0.206) (Fig. 1B, Table 2). Although there were significantly fewer all-cause deaths in the RVP group compared to the CRT group (HR 1.67 (95% CI, 1.16–2.40)) (Table 2, Fig. 2A), there was no significant difference observed between both groups for time to HFH (HR 0.76 (95% CI, 0.45–1.31) (Table 2, Fig. 2B).

### Echocardiographic outcome

**Fig. 3:**
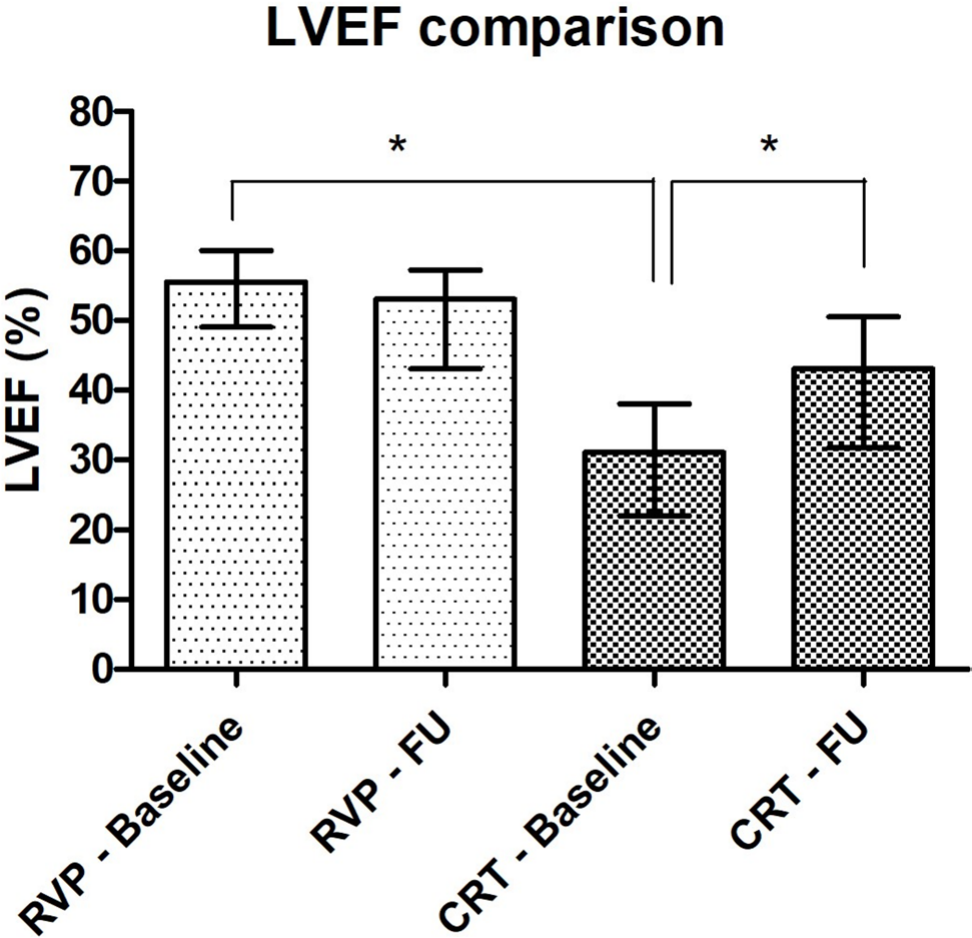
Echocardiography–Median left ventricular ejection fraction (%) with interquartile ranges at baseline and at follow-up in right ventricular pacing group and cardiac resynchronization therapy group. CRT, cardiac resynchronization therapy; FU, follow-up; LVEF, left ventricular ejection fraction; RVP, right ventricular pacing. The asterisk “*” indicates significant difference (*p* < 0.05)

Baseline echocardiographic data within 1 year prior to AVNA and follow-up data were available for 54 patients in the RVP group and for 56 patients in the CRT group. In the RVP group, baseline TTE was performed 152 days [65–245] prior to AVNA, and follow-up TTE was performed 3.4 years [1.2–4.9] after AVNA. In the RVP group, baseline LVEF was 56% [49–60], which did not decrease significantly to an LVEF of 53% [43–57] during follow-up (*p* = 0.081) Fig 3.

During follow-up in the RVP group, 27 patients (50%) experienced a decrease in LVEF, while the other half either remained stable or experienced an increase in LVEF. From these 27 patients, 20 patients (36%) experienced a decrease ≥ 5% in LVEF during follow-up. Seventeen patients (10%) underwent an upgrade to CRT because of worsening HF or PICM. Due to the small amount of available echocardiographic data, no further analyses evaluating possible predictors of LVEF change were performed.

In the CRT group, baseline TTE was performed 126 days [58–197] prior to AVNA, and follow-up TTE was performed 2.9 year [1.1–4.2] after AVNA. Baseline LVEF in this group was 31% [22–38], which significantly increased to a LVEF of 43% [32–51] during follow-up (*p* < 0.001). The baseline LVEF was significantly lower in the CRT group compared to the RVP group (*p* < 0.001) Fig 3.

## Discussion

Long-term follow-up and the optimal pacing modality in the pace-and-ablate strategy remain unclear [3, 12–14]. Several studies investigated the differences between RVP and CRT in the pace-and-ablate strategy but mainly evaluated surrogate (non-clinical) endpoints [12]. In this retrospective, dual-center, observational study, the long-term effects of the pace-and-ablate strategy with either RVP or CRT on clinical outcomes in a large population of patients with AF were investigated. Overall, this study found that all patients treated with the pace-and-ablate strategy were at high risk (48%) for all-cause mortality and HFH (RVP 47% vs CRT 49%). Interestingly, the incidence of HFH did not differ significantly between the two pacing modalities even though the patients in the CRT had significantly lower LVEF at baseline. On the other hand, patients receiving RVP had a lower risk of mortality as compared to patients treated with CRT. In 10% of the patients treated with RVP, a PICM developed necessitating an upgrade to CRT. As the patients in this observational study were treated according to guidelines at that time, and with different indications for CRT as compared to RVP, it is therefore as expected that these groups have different baseline characteristics.

### Clinical outcomes

In our study, all-cause mortality and HFH occurred in 36% and 22%, respectively, of the patients in the RVP group, with a median follow-up of 6.6 years. The mortality rate seems higher when compared to previous studies. Tan et al. reported an all-cause mortality rate of 26%, and Ozcan et al. reported a comparable rate of 22% among patients undergoing RVP after AVNA [14, 15]. However, these studies included younger and less diseased patients, and in the case of Ozcan et al., these had a relatively shorter follow-up period, which likely explains the lower mortality rates compared to our findings [15]. The PAVE study reported an even lower mortality rate of 18% in patients undergoing RVP, although mortality data was not collected as part of an endpoint [16]. Other studies report even shorter follow-up data limited to 6-months [17]. In contrast, comparable incidences of HFH have been observed by Tan et al. and Leclercq et al. who reported a HFH-rate of 20% and 23% for patients undergoing RVP, respectively [14, 17].

Our study showed a 45% mortality rate and a 15% HFH rate in the CRT group, with a median follow-up of 5.2 years. Previous studies have reported dissimilar results with regards to mortality rates [13]. The PAVE study reported a mortality rate of 9% in CRT patients; however, this study randomized AVNA patients to either CRT or RVP, suggesting that some patients in the CRT group may not have a true indication for CRT [16]. This likely resulted in a less diseased CRT population, as also shown by their higher mean baseline LVEF of 47% compared to our median 31%. Furthermore, follow-up only lasted 6 months as opposed to our almost 6 years. The MUSTIC-AF study was not designed as a mortality study and, therefore, does not report separate mortality rates [18]. However the 5-year mortality rate of 27% in the CRT group of this study is consistent with previous literature on HF patients treated with CRT, suggesting that our CRT group is representative of typical CRT patients [19–21]. No comparable studies have specifically evaluated the incidence of HFH in CRT patients treated according to guidelines after AVNA. However, Leyva et al. described a 9% incidence of HF-related hospitalizations within the first year after CRT implantation, not in the context of the pace-and-ablate strategy. This is slightly lower than the incidence reported in this study, which might be explained by the longer follow-up period in this analysis [22].

Interestingly, when comparing HFH incidence between the RVP and the CRT group in this study, no significant difference was found. This indicates that patients undergoing RVP have as many HFH as patients who received CRT. It is important to recognize differences in baseline LVEF which contributes to HFH risk. A meta-analysis by Brandley et al. shows comparable results with no statistically significant advantage concerning HFH for patients receiving CRT when compared to RVP [13]. On the contrary, a meta-analysis by Stavrakis et al. showed that CRT significantly decreased the HFH rate when compared to RVP; however, the statistical significance was lost when one out of three studies was removed from the analysis [12]. This statistically significant decrease in HFH rates can be explained by the fact that in all studies included patients were randomized independent of their LVEF, therefore not treating patients according to the current guidelines (e.g., patients with normal LVEF receiving CRT). Furthermore, the MUSTIC study reported a four times less HFH rate in the CRT group when compared to RVP [18]. However, this extreme difference from our findings can be explained by the study methods of the MUSTIC study. This crossover-trial only included patients with an LVEF < 35%, and a broad QRS complex. This patient population benefits readily from CRT as opposed to RVP [1, 7]. Brignole et al., who compared CRT to RVP in the setting of the pace-and-ablate strategy, found similar results as the MUSTIC study. This study, in contrary to ours, was an RCT with no baseline differences between treatment groups, therefore complicating further comparison to our results [23].

### Cardiac function

The present study demonstrates a significant increase in LVEF following CRT. This improvement in cardiac function can possibly be attributed to the optimized biventricular-pacing rate after AVNA, or to the treatment of TIC as a direct result of the AVNA. The magnitude of LVEF improvement in our study is even greater than previously reported [12, 16]. This might be due to the fact that medical therapy (and guidelines) for heart failure patients have been further improved.

In contrast, the non-significant decrease in LVEF among patients undergoing RVP aligns with previous research [16, 24]. In this study, 10% of patients receiving RVP developed deterioration of LVEF or PICM necessitating an upgrade to CRT. This is lower than what can be found in literature. A systematic review by Somma et al. reported a pooled prevalence of PICM of 12%, with an individual study prevalence range of 6% up to 25%, depending on different risk factors and definitions of PICM [25].

Another observational study by Tops et al. reported a 49% incidence of LV dyssynchrony associated with a decrease in LVEF (48 to 43%) and worsening heart failure symptoms [26]. However, this study included patients with an average baseline LVEF of 48%, thereby placing these patients at a heightened risk of developing PICM. These patients should have been considered for alternative pacing modalities such as CRT.

Thus, pacing-induced deterioration of LVEF remains relatively uncommon. Furthermore, when CRT is used according to guidelines, it significantly improves LVEF in patients treated with the pace-and-ablate strategy.

### Future perspectives

With the introduction of conduction system pacing (CSP) and its ability to provide more physiological stimulation of the ventricles, the threshold for performing the pace-and-ablate strategy has decreased [27]. CSP uses a more physiological approach to pacing, possibly decreasing the risk of PICM. Over the past couple of years, multiple small studies evaluating CSP in the setting of the pace-and-ablate strategy have emerged [28]. These studies show that CSP can lead to an increase in LVEF, a decrease in mortality and an improvement in symptom burden and overall physical fitness when comparing baseline and follow-up measurements. However, most of these studies only include a relatively small population and rarely compare CSP to RVP or CRT in matched cohorts. Studies are needed to investigate the effect of CSP as compared to RVP and CRT in the spectrum of patients with AF managed through a pace-and-ablate strategy.

### Limitations

Due to the retrospective nature of this study and its inherent limitation without baseline randomization, no true causal relation between baseline parameters and outcome variables can be established. Due to the differences in baseline characteristics, this study cannot easily be compared to available RCTs evaluating the differences between RVP and CRT, where baseline characteristics are similar between groups [6, 14, 23].

Furthermore, only a minor portion of all patients had both pre- and post-procedural echocardiographic measurements taken. Due to the lack of a standardized follow-up procedure, only patients with a clinical indication such as history of HF, new onset HF symptoms or worsening of rhythm related symptoms were referred for new TTE after the pace-and-ablate strategy. Therefore, mostly patients with an expected change in cardiac function were included in the analyses, possible leading to an overestimation of the changes in LVEF.

This study found no statistical differences between time-to-first HFH between RVP and CRT. It is crucial to mention that our analysis focused on time-to-first HFH event, which could mask differences in the total number of HFH in either one of the groups. It could be the case that patients in one of the groups might have been hospitalized more than once for HFH, despite similar time-to-first HFH. For instance, there were 17 upgrades from RVP to CRT in this study, which might indicate worsening of HF. However, 12 upgrades (71%) were performed after a first HFH in these patients, not reflected in the time-to-first event analyses.

Since echocardiographic follow-up data and NYHA class are not available for all patients, we cannot determine the number of non-responders to resynchronization therapy. Consequently, we are unable to assess whether this influenced reaching the primary endpoint.

## Conclusions

This study demonstrated that both mortality and HFH rates are high in patients with AF managed through a pace-and-ablate strategy. No significant differences in the occurrence of the primary endpoint were observed when a guideline-directed pacing strategy was implemented. Reassuringly, a deterioration of LVEF requiring an upgrade to CRT is uncommon in patients undergoing RVP who have a normal baseline LVEF prior to AVNA. CRT improves the LVEF in patients with reduced LVEF prior to AVNA.

## Data Availability

The data that support the findings of this study are available from the corresponding author upon reasonable request.
